# Smooth Descent: A ploidy-aware algorithm to improve linkage mapping in the presence of genotyping errors

**DOI:** 10.3389/fgene.2023.1049988

**Published:** 2023-03-01

**Authors:** Alejandro Thérèse Navarro, Peter M. Bourke, Eric van de Weg, Corentin R. Clot, Paul Arens, Richard Finkers, Chris Maliepaard

**Affiliations:** Plant Breeding, Wageningen University & Research, Wageningen, Netherlands

**Keywords:** linkage mapping, genotyping error, identity by descent, imputation, polyploidy

## Abstract

Linkage mapping is an approach to order markers based on recombination events. Mapping algorithms cannot easily handle genotyping errors, which are common in high-throughput genotyping data. To solve this issue, strategies have been developed, aimed mostly at identifying and eliminating these errors. One such strategy is SMOOTH, an iterative algorithm to detect genotyping errors. Unlike other approaches, SMOOTH can also be used to impute the most probable alternative genotypes, but its application is limited to diploid species and to markers heterozygous in only one of the parents. In this study we adapted SMOOTH to expand its use to any marker type and to autopolyploids with the use of identity-by-descent probabilities, naming the updated algorithm Smooth Descent (SD). We applied SD to real and simulated data, showing that in the presence of genotyping errors this method produces better genetic maps in terms of marker order and map length. SD is particularly useful for error rates between 5% and 20% and when error rates are not homogeneous among markers or individuals. With a starting error rate of 10%, SD reduced it to ∼5% in diploids, ∼7% in tetraploids and ∼8.5% in hexaploids. Conversely, the correlation between true and estimated genetic maps increased by 0.03 in tetraploids and by 0.2 in hexaploids, while worsening slightly in diploids (∼0.0011). We also show that the combination of genotype curation and map re-estimation allowed us to obtain better genetic maps while correcting wrong genotypes. We have implemented this algorithm in the R package Smooth Descent.

## Introduction

Linkage mapping is the process by which a set of markers segregating in a population are grouped and ordered. Each marker is placed within a linkage group, oftentimes corresponding to a chromosome, and given a genetic position within that group. The usefulness of genetic mapping has made it a consistent tool during the past century: starting with the study of trait co-segregation in *Drosophila* (Sturtevant, 1913), continuing to the proof of the linear structure of genes and chromosomes (Benzer, 1959), and the first QTL analyses (Lander and Botstein, 1989). Its relevance has not diminished nowadays, as it enables the study of genomic patterns of recombination, thereby highlighting the functional and structural properties of a genome. Linkage maps are also an essential tool for studies in organisms without a reference genome (e.g., (Hu et al., 2021), in plant and animal QTL studies and in the assembly and improvement of genome sequences (Mascher and Stein, 2014; Fierst, 2015).

Genetic mapping algorithms have been greatly influenced by the progress of genotyping. As newer technologies provided larger marker sets, novel mapping algorithms had to be developed to handle growing numbers of markers (Cheema and Dicks, 2009). The most recent genotyping techniques, sequencing-based methods such as genotyping by sequencing (Elshire et al., 2011) or whole genome sequencing (Varshney et al., 2014), are able to identify and genotype millions of variants in a single analysis but suffer from a common drawback: an increased proportion of genotyping errors. That is particularly problematic for the purpose of genetic mapping, since the ordering algorithms on which many mapping approaches rely are notoriously sensitive to errors (Hackett and Broadfoot, 2003; van Os et al., 2005; Cartwright et al., 2007). Since most algorithms depend on pairwise recombination estimates, wrong genotypes can give the false estimate that a double recombination has occurred, producing sub-optimal map orders and inflated map lengths (i.e., >100 cM). The general strategy to deal with this problem has been to detect and eliminate highly spurious markers (Lincoln and Lander, 1992; van Os et al., 2005; Cartwright et al., 2007; Wu et al., 2008; Cheema and Dicks, 2009; Liu et al., 2014; Rastas et al., 2016), although the errors can also be explicitly modelled, increasing the number of retained markers (Bilton et al., 2018).

Polyploidy, the presence of more than two chromosome sets in an organism, is a relatively common condition in crop species (e.g., rose, potato, strawberry, sugarcane, wheat) that poses special challenges in linkage mapping. In autopolyploids, which usually originate from genome duplication within a single species, polysomic segregation and double reduction require specialized methods of linkage estimation (Bourke et al., 2018a). In allopolyploids, arising from interspecific hybrids, segregation usually follows a diploid pattern, but genotyping can be more inaccurate due to the difficulty of distinguishing between homoeologous sequences (Kaur et al., 2012). Although these issues have been addressed with specialized tools and approaches (Glover et al., 2016; Bourke et al., 2018b), these tools were not designed with consideration of the high error proportion in sequencing-based genotype data, and due to the unique challenges of polyploids, diploid-oriented tools cannot be used.

In this study, we aimed to develop a ploidy-aware approach that would help in using high-throughput genotyping information for genetic mapping, without discarding vast amounts of data due to an increased error rate. Therefore, we adapted SMOOTH (van Os et al., 2005), a simple and efficient method for error detection and correction based on the identification of unlikely genotype scores. The original algorithm was only applicable to diploids and to markers heterozygous in only one of the parents. By using identity-by-descent (IBD) probabilities, we extended this model to any ploidy and marker segregation type. Additionally, we changed the k-nearest neighbours approach used in SMOOTH to an interval-based approach, which improves identification and correction of errors in maps with a heterogeneous marker distribution. We term this updated method Smooth Descent, the IBD-based descendent of SMOOTH. Similar to the original algorithm, Smooth Descent requires a preliminary map to be applied, thus it should be thought of as part of an iterative mapping approach, so that with each round of mapping and smoothing a better map is obtained.

This algorithm has been implemented as an R package called ‘Smooth Descent’. The package also generates so-called “graphical genotypes” that can be used as a quality assessment tool by researchers, along with visualizations of the iterative correction process and other diagnostic plots.

## Materials and methods

### Smooth descent approach

SMOOTH and Smooth Descent are both based on the same principle: comparing an observation (error sensitive) and expectation (error tolerant) matrix of genotypes and identifying as errors the inconsistencies between both matrices. The difference lies in the way genotypes are expressed in both approaches: as raw genotype scores in SMOOTH, and as Identity-by-Descent (IBD) probabilities in Smooth Descent. In Smooth Descent observed IBD is obtained through the naive IBD algorithm described below, while expected IBD can be obtained through two methods, weighted average IBD or hidden Markov model IBD. The three methods are described below.

### Naive IBD probabilities

The algorithm begins with parental phasing and a preliminary map that indicates the order and distances of markers. A number of methods can be used, experimental and computational, to obtain parental phasing (Browning and Browning, 2011; He et al., 2018; Al Bkhetan et al., 2021) and a preliminary map (Rastas, 2017; Bilton et al., 2018). In our software, mapping is performed by polymapR (Bourke et al., 2018a) and parental phasing is expected to be obtained by the researcher.

Phased parental genotypes are expressed using the homologue matrix H , in which columns represent parental homologues and rows are markers, ordered according to the preliminary map. The number of columns p will be the sum of parental ploidies. Thus, the matrix H is composed of columns $H_{1}$ to $H_{p}$ . In a diploid cross p=2+2=4 , there would be 4 columns; in a tetraploid cross, 8 and in a cross between a diploid and a tetraploid, 6 columns would be specified. The first set of columns correspond to the homologues of the first parent, and the rest to the homologues of the second parent. Each cell of the H matrix contains a 0 when that homologue holds the reference allele A at that marker, and 1 if it holds the alternative allele B. Because of this, only biallelic markers can be used in Smooth Descent. The choice of reference allele will not influence IBD calculations, and thus it can be done at random. For a diploid cross, an example of H would be:

$$\begin{array}{c} H=\left[\begin{array}{cccc} 1 & 0 & 0 & 0\\ 0 & 1 & 1 & 0\\ 0 & 0 & 0 & 1 \end{array}\right] \end{array}$$ (1)

In a tetraploid:

$$\begin{array}{c} H=\left[\begin{array}{cccccccc} 0 & 1 & 0 & 1 & 0 & 0 & 0 & 0\\ 1 & 0 & 1 & 1 & 1 & 0 & 0 & 0\\ 1 & 0 & 0 & 0 & 0 & 0 & 1 & 1 \end{array}\right] \end{array}$$ (2)

First, we will calculate the error-sensitive, observed IBD probabilities or naïve IBD probabilities. For that we need to obtain all possible homologue combinations that can be inherited, which we denote as *configurations* with the symbol $c_{i}$ . This will depend on the number of homologues that parent 1 and parent 2 pass on to the offspring, which in turn depends on their ploidy.

In the case of a diploid, parent 1 provides a single homologue, either $H_{1}$ or $H_{2}$ ; while parent 2 can provide $H_{3}$ or $H_{4}$ . Although there can be recombinations along the inherited homologues (e.g*.*, switching from $H_{1}$ to $H_{2}$ ), this does not affect our analysis since it is performed marker by marker. Thus, there are four configurations, $c_{1}=\left\{H_{1},H_{3}\right\},c_{2}=\left\{H_{1},H_{4}\right\},c_{3}=\left\{H_{2},H_{3}\right\},c_{4}=\left\{H_{2},H_{4}\right\}$ . On the other hand, in a tetraploid example, each parent will provide two homologues. Thus, a single parent can provide any of six pairs of homologues: $\left(H_{1},H_{2}\right)$ , $\left(H_{1},H_{3}\right)$ , $\left(H_{1},H_{4}\right)$ , $\left(H_{2},H_{3}\right)$ , $\left(H_{2},H_{4}\right)$ or $\left(H_{3},H_{4}\right)$ . Moreover, due to multivalent formation, double reduction scenarios are possible, meaning that parent 1 could also contribute $\left(H_{1},H_{1}\right)$ , $\left(H_{2},H_{2}\right)$ , $\left(H_{3},H_{3}\right)$ or $\left(H_{4},H_{4}\right)$ . If both parents are tetraploid, this amounts to 100 possible configurations. However, since double reduction is relatively rare, and for the sake of simplicity, it has not been considered in this implementation of Smooth Descent. Thus, we will only consider the 36 configurations possible, i.e., we assume that no double recombination occurs.

The next step is to determine the marker *dosage,* $d_{j}$ , (of the alternative allele) of each configuration. This must be calculated independently for each marker. For one marker, matrix H assigns either 0 or 1 to each parental homologue. The inherited dosage of that configuration is simply the sum of the associated parental homologues. For instance, for the first marker (row) in the diploid example, $c_{1}=\left\{H_{1},H_{3}\right\}\;thus\;d_{1}=1+0=1$ while $c_{3}=\left\{H_{2},H_{3}\right\}\;thus\;d_{3}=0+0=0$ . For the first marker of the tetraploid example, $c_{1}=\left\{H_{1},H_{2},H_{5},H_{6}\right\}\;thus\;d_{1}=0+1+0+0=1$ etc.

To obtain IBD probabilities for one individual, one must consider the observed genotype of that individual. Since an individual must hold one of the described configurations, only those configurations whose dosage matches the observed genotype are *possible configurations.* For each genotype g , we denote the set of possible configurations as $C_{g}$ , where $k_{g}$ the number of possible configurations. When no double reduction is considered, all configurations are equally probable, thus the IBD probability of $H_{i}$ is:

$$\begin{array}{c} p\left.\left(H_{i}\right.\right|g)=\frac{\underset{j\in C_{g}}{\sum }f\left(c_{j},H_{i}\right)}{k_{g}} \end{array}$$ (3)

Where $f\left(c_{j},H_{i}\right)$ is an indicator function that takes the value 1 if $H_{i}$ belongs to $c_{j}$ and 0 otherwise.

$$\begin{array}{c} f\left(c_{j},H_{i}\right):\left\{\begin{array}{c} if\;H_{i}\in c_{j}\;then\;1\\ if\;H_{i}\notin c_{j}\;then\;0 \end{array}\right. \end{array}$$ (4)

For example, let us consider an offspring for the two parents represented in the homologue matrix in Eq. 1 with a genotype of 1, 0, 1. The possible inheritance configurations for a diploid parent are $c_{1}=\left\{H_{1},H_{3}\right\},c_{2}=\left\{H_{1},H_{4}\right\},c_{3}=\left\{H_{2},H_{3}\right\},c_{4}=\left\{H_{2},H_{4}\right\}$ . For the first marker $H_{1}=1;H_{2}=0;H_{3}=0;H_{4}=0$ , meaning that each configuration has the following values: c1=1 , $c_{2}=1,$ $c_{3}=0$ and $c_{4}=0$ . Only two configurations, $c_{1}$ and $c_{2}$ are possible given that the genotype is 1, meaning that $k_{g}=2$ . Thus:

$$p\left(H_{1}|1\right)=\frac{f\left(c_{1},H_{1}\right)+f\left(c_{2},H_{1}\right)}{2}=\frac{2}{2}=1$$

$$p\left(H_{2}|1\right)=\frac{f\left(c_{1},H_{2}\right)+f\left(c_{2},H_{2}\right)}{2}=\frac{0}{2}=0$$

$$p\left(H_{3}|1\right)=\frac{f\left(c_{1},H_{3}\right)+f\left(c_{2},H_{3}\right)}{2}=\frac{1}{2}=0.5$$

$$p\left(H_{4}|1\right)=\frac{f\left(c_{1},H_{4}\right)+f\left(c_{2},H_{4}\right)}{2}=\frac{1}{2}=0.5$$

A similar process can be followed for the second marker. In that case $H_{1}=0;H_{2}=1;H_{3}=1;H_{4}=0$ , meaning $c_{1}=1;c_{2}=0;c_{3}=2$ and $c_{4}=1$ . Only one configuration is possible that the genotype is 0: $c_{2}$ , thus $k_{g}=1$ . Applying Eqs 3, 4 as done above yields the following results:

$$p\left(H_{1}|1\right)=1\;p\left(H_{2}|1\right)=0\;p\left(H_{3}|1\right)=0\;p\left(H_{4}|1\right)=1$$

Lastly, the third marker can be computed considering that $H_{1}=0;\;H_{2}=0;H_{3}=0$ and $H_{4}=1$ . Thus, $c_{1}=0;c_{2}=1;c_{3}=0$ and $c_{4}=1$ . In this case the genotype is also 1, meaning that $k_{g}=2$ , since only $c_{2}$ and $c_{4}$ are possible. This yields:

$$p\left(H_{1}|1\right)=0.5\;p\left(H_{2}|1\right)=0.5\;p\left(H_{3}|1\right)=0\;p\left(H_{4}|1\right)=1$$

If we combine these results, we can obtain the IBD matrix $I_{0}$ according to the naive model for this individual:

$$I_{0}=\left[\begin{array}{cccc} 1 & 0 & 0.5 & 0.5\\ 1 & 0 & 0 & 1\\ 0.5 & 0.5 & 0 & 1 \end{array}\right]$$

This algorithm will be applied after each iteration of correction, as described below, to obtain matrix $I_{1}$ , and subsequently to obtain matrix $I_{2}$ , etc.

#### IBD prediction–Weighted average

One of the two methods implemented for IBD prediction in Smooth Descent is based on a local weighted average of observed IBD around a marker, inspired by SMOOTH’s proposal and similar to the procedure suggested by (Wu et al., 2008). This requires two steps: first, defining the set of local markers and second, estimating the weights to be applied to each marker.

Let’s start with marker $m_{i}$ . The set of local markers, $L_{i}$ , are those markers closer than l from $m_{i}$ , where l is a chosen distance threshold (we chose l=10 cM, but a different threshold can be provided). Additionally, low-informative markers will be excluded from the local set. We defined these as markers for which the observed IBD probability is within the 0.3–0.7 range (see Error Prediction section for more information). Since the predicted IBD is calculated per homologue, this means that $L_{i}$ will differ slightly per homologue.

The weight for the observed IBD probability at marker $m_{j}$ will be proportional to the chance that there is no recombination between $m_{i}$ and $m_{j}$ . This no-recombination probability can be obtained from the distance estimates:

$$\begin{array}{c} 1-\rho_{ij}=1-f\left(d_{ij}\right) \end{array}$$ (5)

Where $1-\rho_{ij}$ is the probability of no recombination and $f\left(d_{ij}\right)$ is a reversed mapping function of the distance between $m_{i}$ and $m_{j}$ . Three functions have been implemented: Morgan’s, Haldane’s and Kosambi’s. We can define the weights as:

$$\begin{array}{c} w_{j}=\frac{1-\rho_{ij}}{\sum_{k\in L_{i}}\left(1-\rho_{ij}\right)} \end{array}$$ (6)

For each individual, the predicted IBD probability for marker $m_{i}$ will then be the weighted average of all the markers in $L_{i}$ , for which $d_{ij}<l$ and the observed IBD probability is informative. Applying this along the $I_{0}$ matrix will allow us to calculate the predicted IBD matrix $\hat{I_{0}}$ .

#### IBD prediction–Hidden markov model

The second model for IBD prediction is based on a hidden Markov model (HMM), a common approach to obtain error-tolerant IBD estimates (Zheng et al., 2016; Mollinari and Garcia, 2019; Zheng et al., 2021). We have included in Smooth Descent the HMM implemented within polyqtlR (Bourke et al., 2021), an expanded version of the TetraOrigin model (Zheng et al., 2016). This HMM uses a discrete-time Markov chain to model parental origins of chromosomes along the markers of each offspring. To do so, it models homologue pairing in the gamete’s meiosis, including recombination probabilities and gamete fusion to constitute a zygote, thus closely modelling the biological reality of inheritance. By defining a series of likelihoods for the parental haplotypes conditional on the offspring genotypes, it provides a powerful tool for estimating IBD probabilities and recombination points.

#### Error prediction

In SD error estimation is performed by comparing an error-sensitive IBD matrix (naive IBD) with an error-tolerant matrix (weighted average IBD, or HMM IBD). Therefore, using SD one can obtain error estimates by comparing naïve probabilities to the weighted average probabilities, or to the HMM-based IBD probabilities.

Each IBD matrix, $I_{0}$ or $\hat{I_{0}}$ is composed of IBD probabilities for each homologue and each marker, which we term $i_{0}$ and $\hat{i_{0}}$ respectively. The principle of error prediction is to identify markers for which their observed and predicted IBD probabilities disagree strongly, meaning that the observed genotype clearly indicates a homologue inheritance that does not match the predicted IBD. More formally, an error can be identified if $\left|i_{0}-\hat{i_{0}}\right|>\delta$ , where δ is an error threshold preferably above 0.7.

Due to this definition, low-informative markers (with observed probabilities between 0.3 and 0.7) must be excluded from the weighted-average IBD prediction step. The contrast $\left|i_{0}-\hat{i_{0}}\right|$ will not reach a high value if either $i_{0}$ or $\hat{i_{0}}$ are close to 0.5. The observed IBD $i_{0}$ will be close to 0.5 if the observed inheritance is uncertain, which means we do not have enough information to discern whether that genotype is an error. The predicted IBD $\hat{i_{0}}$ , should be close to 0.5 if the set of local markers have both high and low IBD probabilities, indicating that there is a local disagreement on inheritance. If low-informative markers are kept, even if many informative markers exist that clearly indicate homologue inheritance, the presence of low-informative markers will centralize the local weighted average and prevent identification of putative errors. Thus, low-informative markers should be removed from IBD prediction.

#### Genotype correction and iteration

When a marker is detected as erroneous, a new genotype can be imputed by computing the most likely marker genotype according to the predicted IBD. The new set of genotypes can be used to calculate an improved map, and a corrected IBD matrix, $I_{1}$ . The previous steps can then be repeated to obtain a new error matrix $E_{1}$ and further improved genotypes. Thus, an iterative approach emerges, where in each iteration the genotypes are further corrected. As iterations progress the genetic map is expected to change less, and thus we are more certain of the achieved order. In view of caution regarding the introduction of artefacts, the error threshold was set at δ=0.9 during the first iteration, and then slowly decreased to 0.7 as iterations progress.

#### Best iteration selection

When using Smooth Descent, we must choose the best iteration according to some criterion. We offer the $R^{2}$ estimate of the second-order polynomial relationship (i.e*.*, $d=a+br+{cr}^{2}+\varepsilon$ ) between inter-marker distance d , and the recombination frequency r (not to be confused with distance-based recombination frequency ρ used for IBD prediction). Unlike ρ , r is calculated during the mapping process through a likelihood or Bayesian method and is the basis of the final map order. In a good map, the relationship between $r_{ij}$ and $d_{ij}$ should be mostly linear, where high recombination frequencies lead to high distances. Thus, the iteration with the highest $R^{2}$ can be considered the best.

### Simulated data

PedigreeSim (Voorrips and Maliepaard, 2012), a program that simulates meiotic pairing and recombination for a range of pedigrees and ploidies, was used to simulate genotype data. We simulated diploids, tetraploids and hexaploids. For each ploidy, ten F1 populations were simulated (30 in total) with 100 individuals each. Every individual had one single chromosome containing 200 segregating markers distributed at variable densities along the chromosome. Error rates were applied randomly by changing the genotypes of 1%, 5%, 10%, 20% of the markers.

Additionally, two special cases were designed to test the effect of variable error rates across individuals (special case A) and across markers (special case B). Special case A contained 80 individuals with an error rate of 0.02 and 20 individuals with an error rate of 0.3. Special case B had the same error rate for all individuals, but variable across markers, ranging in a continuous curve along the chromosomes. The curve was defined as a smooth spline passing through the error rates 0.02, 0.1, 0.3, 0.02 and 0.1 at approximately 25 cM intervals along the chromosome. Thus, high error rate markers were located close to one another and at the centre of the chromosome.

Each genotype dataset was mapped using Smooth Descent with 10 iterations and tested using the weighted average or HMM method for computing error-tolerant IBD probabilities. To evaluate the effectiveness of SD, as well as the additional tools tested, three parameters were used: genotyping error, the percentage of genotypes different from the true genotypes; position correlation, the correlation between the true map positions and estimated map positions; and map length, the size of the estimated genetic map.

### Real data

Data from strawberry (*Fragaria* x *ananassa*) data was obtained from whole genome sequencing of 48 individuals from an F1 population. Variant discovery was performed using bcftools and genotyping with the R package “updog” (Gerard et al., 2018)^,^ allowing to genotype ∼10 M markers. After filtering markers based on depth and genotyping quality, ∼1.8 M markers were kept and summarised into ∼6,500 unique markers across all chromosomes. Due to a skim sequencing strategy, many genotyping errors were expected and observed, which proved this dataset useful for testing our approach. Since strawberry is an allopolyploid with strict chromosomal pairing behaviour, the data could be treated as that of a diploid.

Data from sweet potato (*Ipomoea batatas*) was taken from (Mollinari et al., 2020). Sequencing was performed using the polyploidy-optimized method described in GBSpoly (Wadl et al., 2018). The obtained read counts were passed to SuperMASSA (Serang et al., 2012) and genotypes were filtered for quality. For chromosome 15, a final count of 1,513 genotypes were obtained for 287 individuals. These genotypes were used with SD, creating a preliminary map *de novo* and performing genotype correction on the genotypes. A single iteration of SD was used since no more improvements could be made subsequently.

Data from diploid potato was taken from (Clot et al., 2022). The dataset consisted of 1,536 full-sibs from a cross between two heterozygous clones C (USW5337.3) and E (77.2102.37). This population was skim sequenced to an average coverage of ∼1.5x. Parent specific SNPs were called using bcftools v.1.13 and used to impute haplotypes in bins of 0.1 Mbp resulting in 4,893 female and 4,735 male segregating markers. Smooth Decent was used based on physical position with five rounds at prediction interval of 1 Mbp and two final rounds with a prediction intervals of 5 and 10 Mbp respectively.

### Software comparison

SD is a unique tool since it is the only available tool that aims at correcting polyploid (and diploid) linkage maps while simultaneously correcting genotyping errors. However, other tools exist that can perform one of the two functions. We have compared SD to polymapR (Bourke et al., 2018a), a polyploid linkage mapping approach that does not perform genotype correction; and to MAPpoly (Mollinari and Garcia, 2019), a HMM approach that is able to correct genotypes and re-estimate marker positions but that does not re-compute linkage map orders.

Ten F1 populations equivalent to those described in the Simulated Data section were used. Genotyping errors were added at a rate of 1%, 5%, 10%, 15%, 20%, 25% and 30%. For each population and error rate four approaches were tested: polymapR, MAPpoly, SD using weighted average IBD prediction and SD with HMM IBD prediction. For both MAPpoly and SD the same preliminary map was provided. Additionally, the error prior provided to MAPpoly was the actual simulated error rate. Lastly, SD results were obtained with 5 iterations since previous results (see Simulation results) showed that iterating more than 5 times did not have a significant impact in the result.

After running each approach, position correlation (correlation between true and estimated map positions), map length and computational run-time were obtained. Genotyping error was only calculated for SD and MAPpoly methods, since polymapR does not perform genotype correction.

## Results

### Simulated data

**FIGURE 1:**
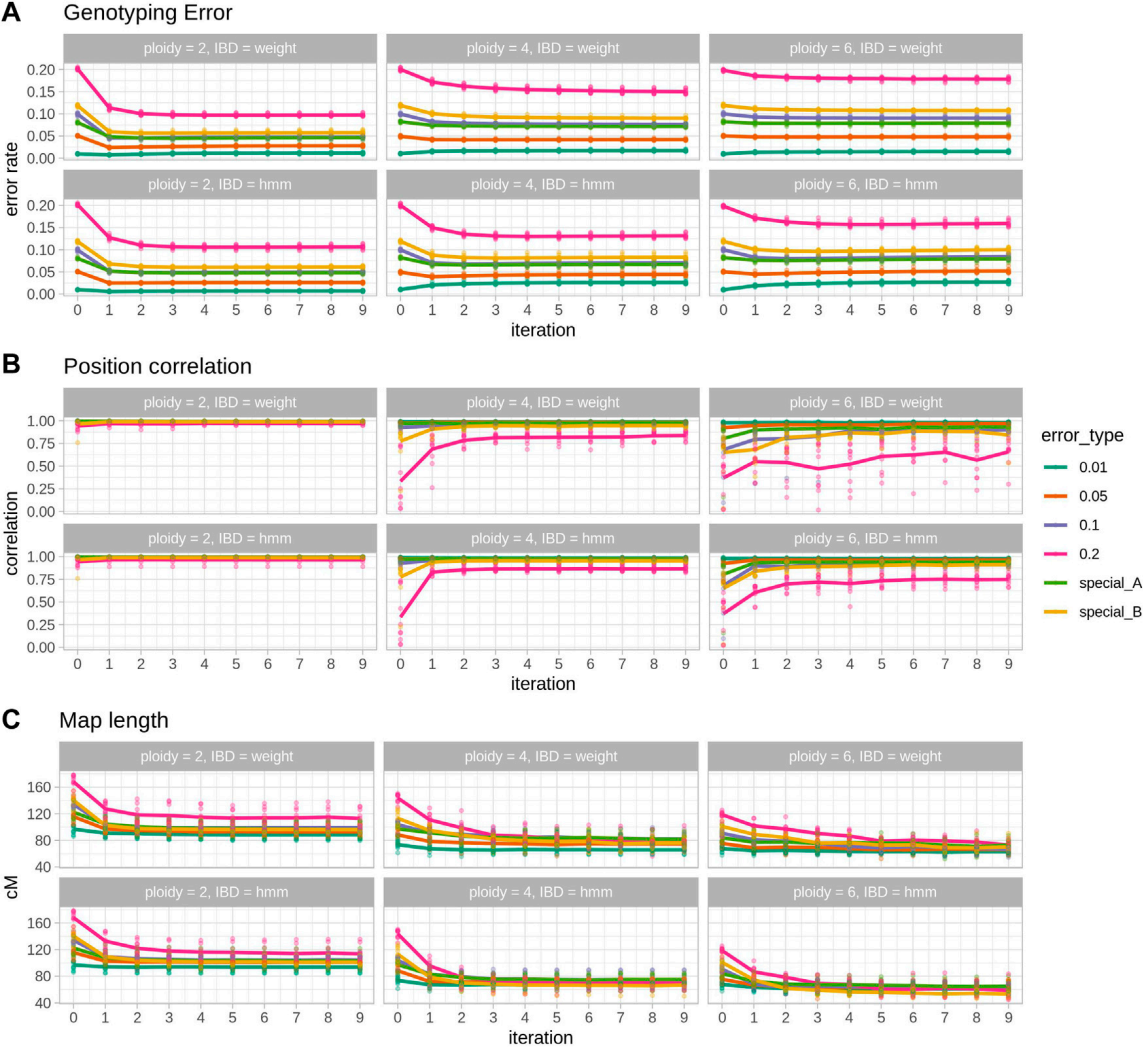
Results of 10 simulated populations across error rates and ploidies. Within each section, each column represents a ploidy and panel the top row shows the results for the IBD estimation with the weighted average procedure (IBD = weight) and the bottom row for the IBD estimation with the HMM (IBD = hmm). **(A)** Genotyping error, the rate of genotypes that are different from the true genotypes. **(B)** Position correlation, the correlation between true genetic positions and estimated positions in a genetic map. **(C)** Map length, the size in cM of the estimated maps. In each plot, points represent individual observations and lines are the average. Each colour represents one simulated error type, with special A being heterogeneous rate across individuals and special B being heterogeneous rate along the map.

A total of 10 populations per ploidy were tested with 6 different levels of genotyping error and two IBD prediction methods, showing the usefulness of Smooth Descent (SD) in correcting genotypes, improving map orders and shortening map lengths (Figure 1). It can be observed how the most impactful changes occur in the first few iterations: the biggest change in genotype correctness (Figure 1 top), the largest improvement in genetic map correctness (Figure 1 middle) and the biggest reduction in map length (Figure. 1 bottom). Note that map length was particularly short in polyploids (∼60 cM in tetraploids and ∼45 cM in hexaploids), an issue that seems to stem from preliminary map calculation.

Ploidy is an important factor in the behaviour of SD, moving from a genotype corrector at lower ploidies to a map corrector in higher ploidies. In diploid cases (Figure 1 left column) SD is able to halve genotyping error (e.g. ∼5% reduction in the 0.1 error rate scenario, Figure 1 left top; Table 1) and to shorten map lengths, especially in the highest error rate cases (e.g., ∼30 cM shortening, Figure 1 left bottom, Table 1). Nevertheless, in diploids, SD does not significantly impact the correlation (there’s a small decrease) between true and estimated map positions, since the preliminary map is already highly correlated to the true map, although longer. In contrast, in polyploid scenarios reduction of genotyping error is smaller (Table 1; Figure 1 middle and right columns), but the correlation between true and estimated maps improves substantially, especially in the hexaploid case. Map size reduction is of the same order, about 30 cM. Importantly, for lower error rate cases, there was a slight increase in genotyping errors, although this did not affect the correlation with the true map or map size. This can be attributed to incorrect imputations by the SD algorithm. Wrong imputations occur in all scenarios, but in most cases they represent a small fraction of the imputed genotypes, finally yielding an overall improved genotype correctness. Only when ploidy is high and genotyping error is low the number of correct genotypes decreases due to wrong imputations.

**TABLE 1:** Average change between preliminary map and last iteration of Smooth Descent.

Error rate (%)	Ploidy	IBD method	Δ Error (%)	Δ Correlation	Δ Size (cM)
*1*	*2*	*hmm*	−0.27	−0.0008	−3.10
*1*	*2*	*weight*	0.16	−0.0032	−8.27
*1*	*4*	*hmm*	1.58	−0.0034	−6.05
*1*	*4*	*weight*	0.69	−0.0013	−7.94
*1*	*6*	*hmm*	1.76	−0.0031	−7.12
*1*	*6*	*weight*	0.53	0.0014	−4.70
*10*	*2*	*hmm*	−4.98	−0.0011	−29.41
*10*	*2*	*weight*	−5.15	−0.0020	−35.01
*10*	*4*	*hmm*	−2.94	0.0302	−29.00
*10*	*4*	*weight*	−2.33	0.0298	−22.44
*10*	*6*	*hmm*	−1.51	0.2354	−30.88
*10*	*6*	*weight*	−0.95	0.2083	−24.64

Two error rate cases (0.01 and 0.1) are shown to illustrate the difference between the last iteration of SD and the preliminary error rate (Δ Error), correlation between the true map positions and estimated map positions (Δ Correlation) and map size (Δ Size). All values were calculated as last iteration–preliminary value (positive means increase, negative means decrease). Values are shown for all ploidies and IBD estimation methods (hmm is hidden Markov model and weight is weighted average method).

The two IBD prediction methods tested (weighted average and HMM) performed similarly in diploids but had some differences as ploidy increased. Genotyping error correction was better for the HMM as ploidy and initial error rate increased (Table 1, error rate 10%). Consequently, estimated map positions and map sizes were also better for the HMM in high ploidy and high error rate cases. However, at lower error rates the HMM method produced a larger increase in genotyping errors (Table 1, error rate 1%).

### Real data

**FIGURE 2:**
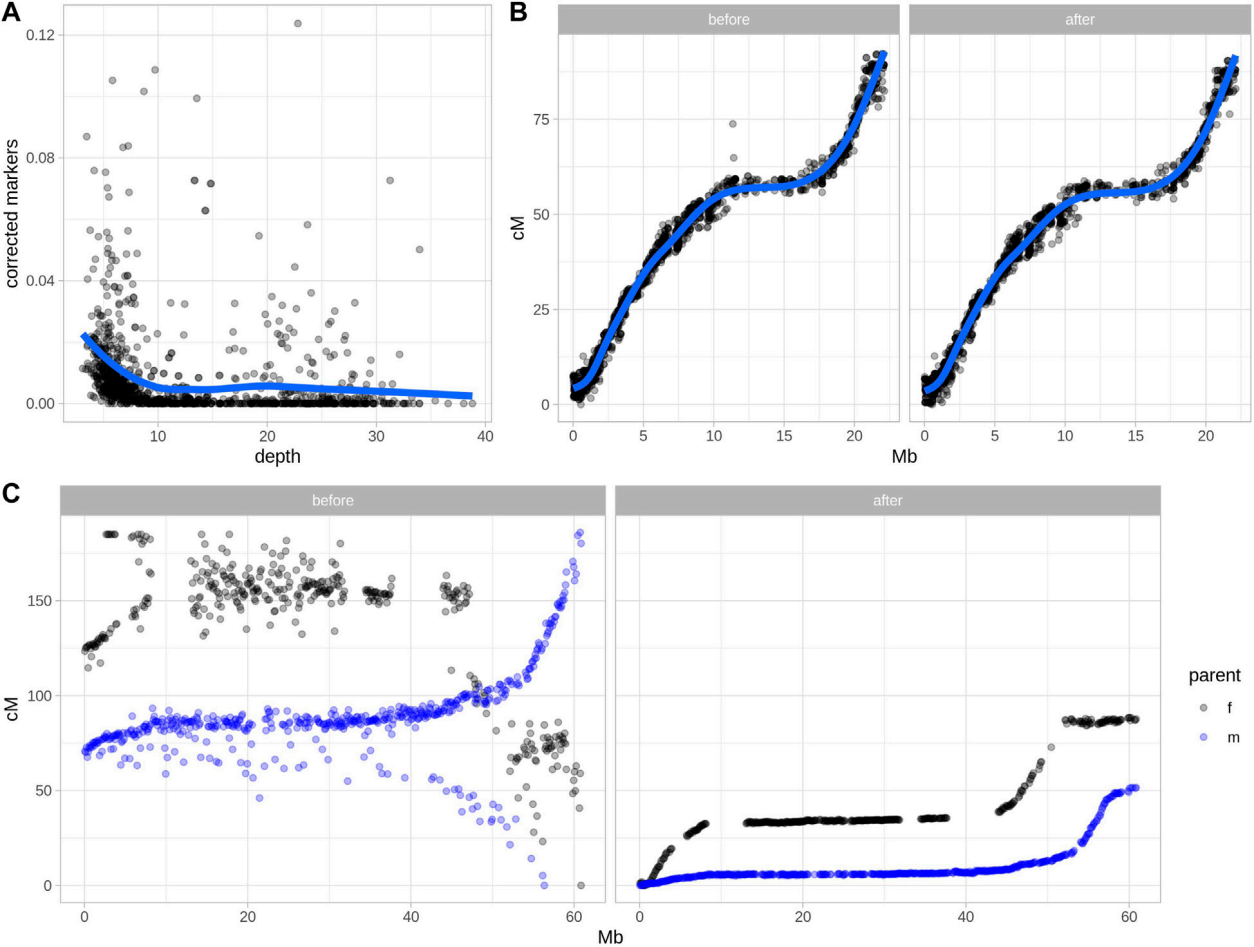
Error detection and marker order in two real datasets after applying Smooth Descent. **(A)** Relationship between sequencing depth and the rate of markers corrected by Smooth Descent for each chromosome of 52 individuals of strawberry (*Fragaria x ananassa*). **(B)** Relationship between physical and genetic positions of 1,513 markers in chromosome 15 of *Ipomoea batatas,* before and after correcting 7.38% of genotype calls using Smooth Descent. **(C)** Relationship between physical and genetic positions of 1716 markers in chromosome 12 of *Solanum tuberosum,* before and after using Smooth Descent to correct low depth genotypes based on a physical order.

Two real datasets were tested using Smooth Descent, a low-depth dataset of garden strawberry (*Fragaria x ananassa*) (Figure 2A), chromosome 15 of *Ipomoea batatas* (Figure 2B) and a low-depth dataset of a diploid potato (Figure 2C)*.* Each strawberry chromosome was mapped using a relatively small population genotyped at low depth. Smooth Descent corrected up to 13% of genotypes, largely correlating with depth so that samples sequenced at lower depth had more genotype corrections. About 3.5% of studied chromosomes had a depth above 10x and had more than 2% of genotypes corrected, an unexpected result probably caused by errors during mapping leading to overcorrection of some samples.

The dataset of autohexaploid *I. batatas* was used to test SD in a scenario with better genotype accuracy. SD corrected 7.38% of genotypes while maintaining an equivalent relationship between the physical and genetic maps (Figure 2B). This highlights the ability of SD to improve genotype accuracy even in situations where there have not been major issues in defining linkage map.

Lastly, a diploid dataset of potato was genotyped using very low sequencing coverage of ∼1.5x, which suggested a low-quality genotypic dataset (Clot et al., 2022). Separate parental maps were generated and each group of markers was corrected using SD with physical order as an input, since a high-quality potato genome sequence was available. The results show a drastic improvement in the correlation between the physical and genetic maps before and after applying Smooth Descent.

### Software comparison

The performance of Smooth Descent was compared to two similar software tools: polymapR (Bourke et al., 2018a) and MAPpoly (Mollinari and Garcia, 2019). The former performs linkage mapping in polyploids without considering genotyping errors. The latter uses a pre-determined order and a HMM method to obtain new map distances and new genotypes.

**FIGURE 3:**
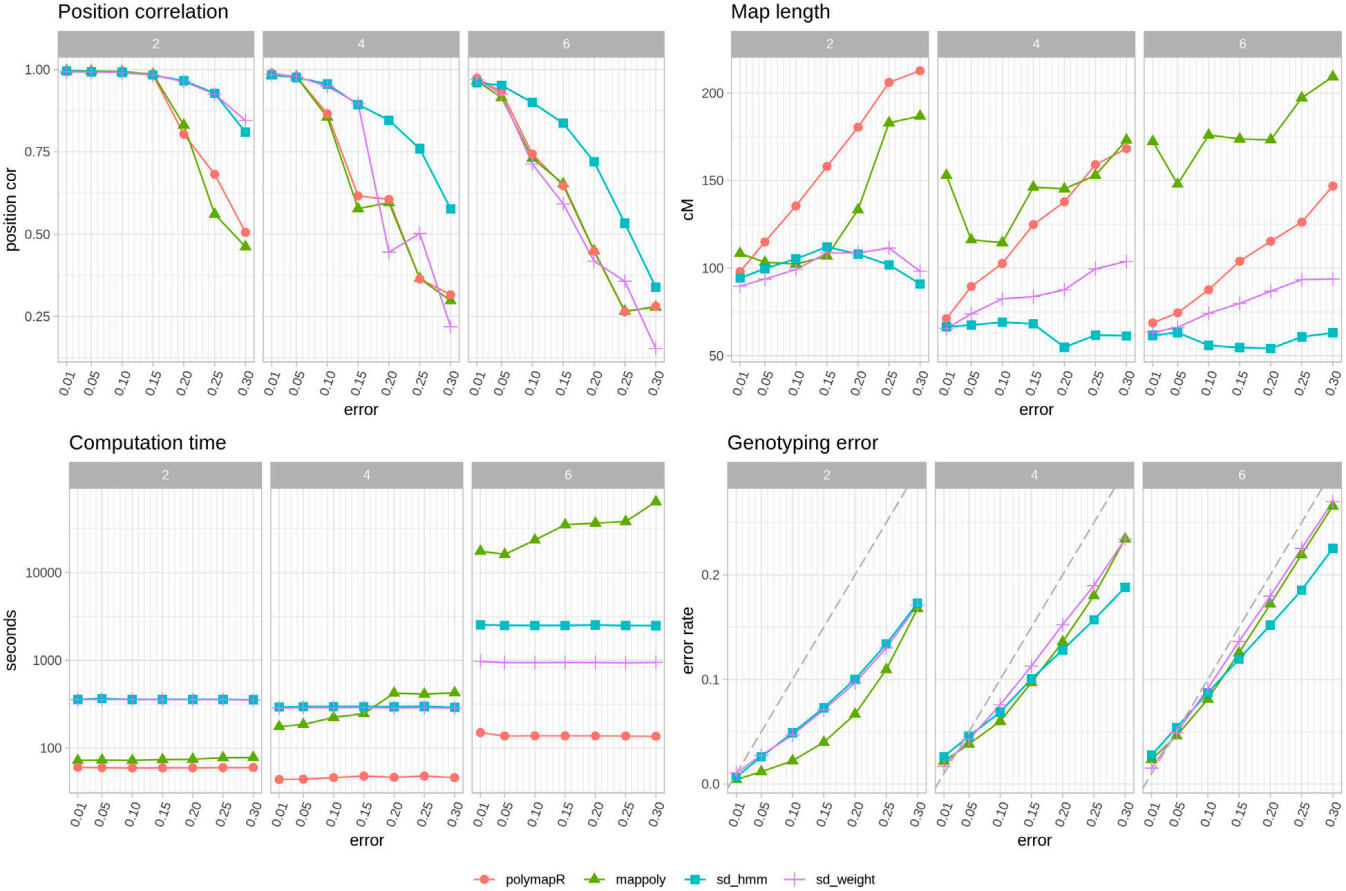
Software comparison with other tools. Average observations of 10 populations per ploidy with simulated genotyping errors. Top left, correlation between true map position and estimated map position. Top right, map length in cM. Bottom left, time spent in seconds, note logarithmic y axis. Bottom right, genotyping error of corrected genotypes. Grey dashed line indicates the starting error rate. Note that polymapR does not produce corrected genotypes and thus is not included in this panel. Each color and shape corresponds to a different approach: red circle, polymapR, green triangle MAPpoly, blue square (sd hmm) SD with HMM approach, purple cross (s _weight) SD with weighted average approach.

In Figure 3 we can see the improvements that SD brings. The reconstructed maps have better position correlation and shorter lengths with SD, particularly when the error rates increase. Importantly, only SD changes the order as genotyping errors are corrected, a feature that is clearly useful especially as the error rate and ploidy increases (Figure 3 top left). As expected, higher error rates lead to longer maps when using polymapR, but surprisingly, in MAPpoly that is the case with both very low or very high error rates. Note that polyploid map lengths are much shorter than expected, an issue that is common to polymapR and SD. In terms of genotyping error correction, MAPpoly is better than SD in diploids, but both perform equivalently well in polyploids, except in higher error rates where the HMM of SD is somewhat better. Lastly, the computation time needed for 5 iterations of SD is around 400 s in diploids and tetraploids, and around 1000 s or 2500 s in hexaploids for the weighted average or HMM methods. In comparison, polymapR was always faster, which is to be expected since SD is iteratively running polymapR. MAPpoly time consumption was much higher as ploidy and error rate increased, with very long waiting times in hexaploids.

Overall, SD is better at recovering the correct order and shortening maps regardless of the situation. MAPpoly was better in the diploid scenario in terms of genotype correction and time consumption but became equivalent or worse than SD in tetraploids and hexaploids.

## Discussion

In this study we have shown that Smooth Descent is able to substantially reduce genotyping errors, particularly in diploids, and to greatly improve marker order in polyploid linkage mapping, especially using the HMM approach. Moreover, when compared to related tools, SD computes better linkage maps with an equivalent or better level of genotype correction. Our findings are supported by analysis of real data: there was a clear correlation between sequencing depth and estimated genotyping errors in a low-depth strawberry dataset, and an accurate genetic map was obtained after correcting around 7.4% of genotyping errors in hexaploid sweet potato. Thus, we have shown that genotype correction is a useful method to improve linkage mapping in the presence of genotyping errors.

In contrast, the most popular strategy of error management in current genetic mapping software is marker or genotype removal. In JoinMap this is achieved through a Bayesian parameter (Liu et al., 2014), while Lep-Map2 does so through a Hidden Markov Model (HMM) (Broman et al., 2003; Rastas et al., 2016). GUSMap, on the other hand, does not remove errors but compensates their impact in map length, also within an HMM model (Bilton et al., 2018). Finally, HighMap uses SD’s predecessor SMOOTH (van Os et al., 2005), and thus could benefit from the developments presented here (Liu et al., 2014).

The genotype correction approach presented in this article depends on transmitting confident parental information to uncertain offspring genotypes. Essentially, if most local markers indicate that one chromosomal region of a parent has been inherited, the offspring genotypes should match parental haplotypes. This rationale, and therefore the accuracy of SD, depends on two important factors: marker order and parental phasing.

### Marker order

The set of local markers used to identify wrong genotypes is clearly defined by marker order. It is not crucial that marker order is exact, but the overall preliminary order should be correlated to the true order. In instances where the provided preliminary order is very far off from the true order, SD will not be able to impute genotypes correctly and any map improvement will be spurious.

Marker order can be determined by a linkage mapping procedure where a measure of linkage and an ordering algorithm is used to obtain a genetic map. In our implementation of SD these correspond to polymapR (Bourke et al., 2018a) and MDSMap (Preedy and Hackett, 2016) respectively. Both processes are sensitive to genotyping errors, meaning that as errors increase, the accuracy of the estimated linkage map will decrease. Consequently, there is a natural upper limit to the level of genotyping error that SD can tolerate: once the error rate impedes the calculation of a relatively good preliminary genetic map, SD stops being useful. This also means that if different methods were designed that could compute marker orders independently of genotyping errors, SD applicability would be expanded.

Linkage mapping is not the only way to determine marker order. As reference genomes are built, it is increasingly common to obtain physical positions for markers. If such information is available, one could apply SD using physical, instead of genetic positions. This opens the possibility of using SD to datasets that are too large to be mapped using linkage techniques, but that could benefit from an error-cleaning algorithm. Moreover, since the order would not need to be re-calculated after genotype correction, only a single iteration of the algorithm would be necessary. Nevertheless, particularly for the weighted-average IBD estimation procedure, the usage of physical positions rather than genetic positions could be problematic since physical distances do not represent the same recombination probabilities along the genome. In centromeres a distance of 100,000 bp will include less recombinations than 100,000 bp in the chromosome arms. This should not be a major problem in the application of SD though, since the recombination frequencies are used relative to each other within small local intervals. Furthermore, if the locations of the pericentromeric regions are known (which they often are), then it would be possible to generate pseudo-cM positions of markers to circumvent this issue.

### Parental phasing

To calculate identity-by-descent (IBD) probabilities, the backbone of genotype error detection and correction in SD, accurate parental phases or parental haplotypes are required. In this study we have not aimed at characterizing the effects of parental phasing in SD, as there has been much research dedicated to this complex issue (Browning and Browning, 2011; He et al., 2018; Al Bkhetan et al., 2021), both in diploids and in polyploids. Currently, there are two types of approaches that can be used to establish parental phasing: based on marker scores or on sequence reads.

Marker scores have been used within several Hidden Markov Models (HMM) to obtain accurate phases. Recent studies in diploid data showed that consensus haplotyping approaches are the most accurate (Al Bkhetan et al., 2021), although individually tools like SHAPEIT4 (Delaneau et al., 2019) and BEAGLE5 (Browning et al., 2018) have the best performances in terms of time efficiency and accuracy. Several HMM have also been developed focused on polyploid data which can estimate phases: MAPpoly (Mollinari and Garcia, 2019), polyOrigin (Zheng et al., 2021), and polyqtlR (Bourke et al., 2021). Although many of these methods consider genotyping error in their estimations, since phasing depends on marker segregation, an increased genotyping error rate in the target population can decrease phasing accuracy.

Alternatively, reads can be used to perform haplotype assembly: by observing multiple polymorphisms in a single read one can infer the most likely haplotype phases. Multiple tools have been developed to produce long-range haplotypes using short reads, long reads or a combination of both (Garg, 2021). In diploids, WhatsHap (Patterson et al., 2015) and HapCut2 (Edge et al., 2017) are the most popular methods, being able to produce chromosome-level haplotypes when combining short and long read data (Garg, 2021). In polyploids, the assembly problem is more complex, which has required the development of specific tools such as HapCompass (Aguiar and Istrail, 2012), HapTree (Berger et al., 2014) and SDhaP (Das and Vikalo, 2015). Although useful, the accuracy of these tools is quite variable depending on depth and ploidy (Motazedi et al., 2017), never reaching the performance of their diploid counterparts. More recent developments like WhatsHap polyphase (Schrinner et al., 2020), based on long-read sequencing or Hap10 (Majidian et al., 2020), oriented to link-read data, are promising in closing the gap between diploid and polyploid haplotype assembly.

### Application of Smooth Descent

The original idea behind the development of SD was to create a tool that would be able to utilize low-depth, inaccurate genotypes to obtain accurate linkage maps. Intuitively, we expected that confident parental phasing would be enough to create such an approach. We have shown that indeed, if parental information is accurate and marker order is well established, genotype correction can be performed, and accurate linkage maps obtained. Thus, we can imagine the following genotyping setup for an F1 population. First, the two parents are sequenced at high depth using long-read sequencing, in order to compute parental haplotype phases. Secondly, the F1 population is genotyped using low-depth short reads. If a marker order is not established yet, SD can be used iteratively to improve genotypes and obtain an accurate linkage map. Otherwise, a single iteration of SD is used to eliminate as many genotyping errors as possible. If the marker number is relatively small, the HMM method of SD is applied, if the dataset is larger the more efficient, although less accurate, weighted average method is used. Finally, a set of corrected genotypes is obtained. In this manner, SD would reduce genotyping costs by allowing a lower depth of sequencing in the F1 offspring.

Overall, SD is a simple and informative software tool. It estimates IBDs, calculates error rates per marker and individual and can impute corrected genotypes. Our implementation, together with MDSmap (Preedy and Hackett, 2016) and polymapR (Bourke et al., 2018a) allows SD to work in multiple ploidies and with large datasets. We also provide many visualization tools which will help uncover the hidden information within genotyping data and turn Smooth Descent into SMOOTH’s descendent.

## Data Availability

The datasets presented in this study can be found in online repositories. The names of the repository/repositories and accession number(s) can be found below: https://figshare.com/, https://figshare.com/articles/dataset/Smooth_Descent_results/20038589.
