# Supplementary figures and images for: Smooth Descent: A ploidy-aware algorithm to improve linkage mapping in the presence of genotyping errors
Source: Front Genet. 2023 Mar 1;14:1049988. doi: 10.3389/fgene.2023.1049988 (PMC10014611; doi:10.3389/fgene.2023.1049988)

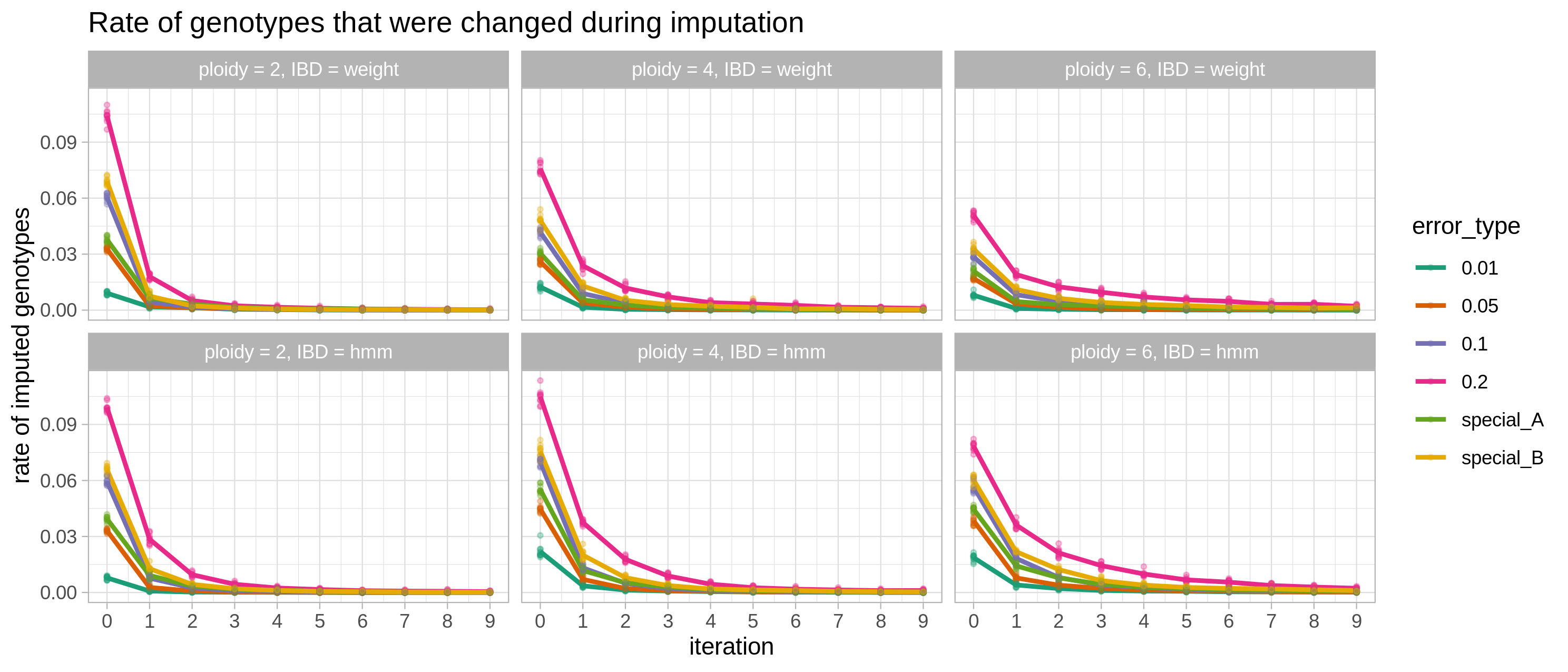

Supplement: Supplementary file 1 [file Image2.PNG]

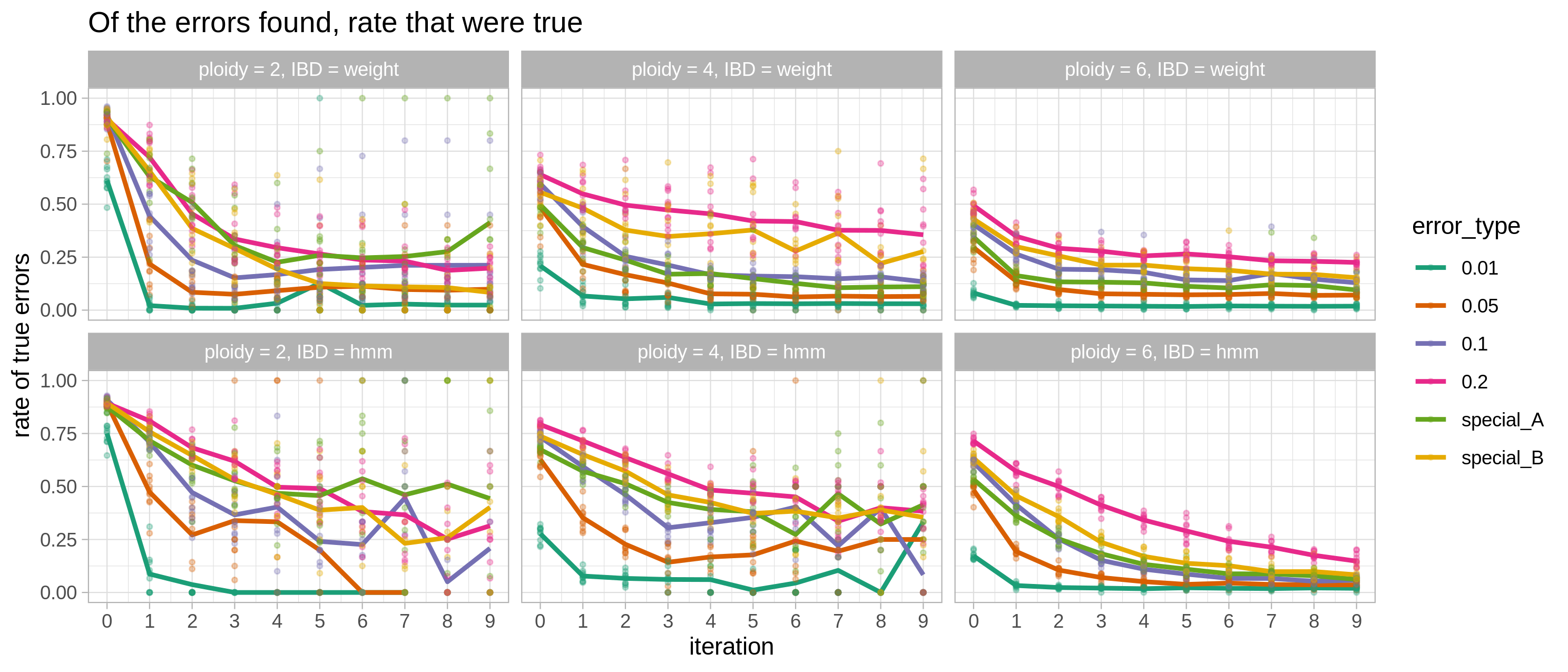

Supplement: Supplementary file 2 [file Image1.PNG]
